# Cancer Cell Glycocalyx and Its Significance in Cancer Progression

**DOI:** 10.3390/ijms19092484

**Published:** 2018-08-22

**Authors:** Hongyan Kang, Qiuhong Wu, Anqiang Sun, Xiao Liu, Yubo Fan, Xiaoyan Deng

**Affiliations:** 1 Key Laboratory for Biomechanics and Mechanobiology of Ministry of Education, School of Biological Science and Medical Engineering, Beihang University, Beijing 100083, China; hongyankang@buaa.edu.cn (H.K.); didada2188@163.com (Q.W.); saq@buaa.edu.cn (A.S.); liuxiao@buaa.edu.cn (X.L.); 2 Beijing Advanced Innovation Centre for Biomedical Engineering, Beihang University, Beijing 102402, China; 3 National Research Center for Rehabilitation Technical Aids, Beijing 100176, China

**Keywords:** glycocalyx, cancer, mechanotransduction

## Abstract

Cancer is a malignant tumor that threatens the health of human beings, and has become the leading cause of death in urban and rural residents in China. The glycocalyx is a layer of multifunctional glycans that covers the surfaces of a variety of cells, including vascular endothelial cells, smooth muscle cells, stem cells, epithelial, osteocytes, as well as cancer cells. The glycosylation and syndecan of cancer cell glycocalyx are unique. However, heparan sulfate (HS), hyaluronic acid (HA), and syndecan are all closely associated with the processes of cancer progression, including cell migration and metastasis, tumor cell adhesion, tumorigenesis, and tumor growth. The possible underlying mechanisms may be the interruption of its barrier function, its radical role in growth factor storage, signaling, and mechanotransduction. In the later sections, we discuss glycocalyx targeting therapeutic approaches reported in animal and clinical experiments. The study concludes that cancer cells’ glycocalyx and its role in cancer progression are beginning to be known by more groups, and future studies should pay more attention to its mechanotransduction of interstitial flow-induced shear stress, seeking promising therapeutic targets with less toxicity but more specificity.

## 1. Introduction and overview

The glycocalyx is a surface layer that covers multiple cells (i.e., endothelial cells, smooth muscle cells, stem cells, and cancer cells, among others) and is mainly composed of proteoglycans and glycoproteins. The composition, physiology, and pathology of vascular cell glycocalyx have been sophisticatedly reviewed in several published papers. In the present review, we attempt to elucidate knowledge about cancer cell-specific glycocalyx: Its altered glycosylation and syndecan expression. Principle emphasis is on the effects of different components of the glycocalyx (heparan sulfate, hyaluronic acid, syndecans) on the progression of cancer, including the convenience of cancer cell migration and metastasis, cancer cell adhesion, tumorigenesis and tumor growth. We also discuss the possible mechanisms of glycocalyx involved in cancer progression and collate glycocalyx-specific targeting therapeutic approaches that have been reported up to now. 

## 2. The Glycocalyx

### 2.1. Glycocalyx in General

The glycocalyx (GCX) is a multifunctional layer of glycans that presents on the surface of cardiovascular cells, cancer cells, red blood cells, gut cells and ocular surface. A toolkit of genetically encoded glycoproteins and expression systems to manipulate the structure and composition of the cellular glycocalyx was recently developed by Shurer [1] and his team.

**Figure 1:**
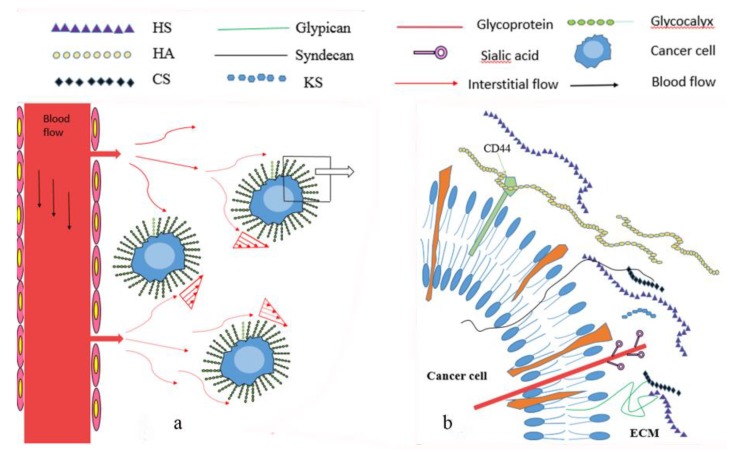
(**a**) Cancer cells are exposed to interstitial flow and glycocalyx can sense interstitial flow induced shear stress. (**b**) Glycocalyx is composed of proteoglycans and glycoproteins, like HS, HA, CS and KS. Syndecans and glypicans are the major core proteins.

Glycocalyx is mainly composed of proteoglycans and glycoproteins (Figure 1). Proteoglycans are formed by the covalent attachment of a core protein with one or more glycosaminoglycan (GAG) chains through serine residues [2]. GAGs are long linear, acidic carbohydrates polymers with repeating disaccharide units, which are strong negatively charged and hydrophilic. GAGs can be divided into the following four major categories: Heparan sulfate/heparin (HS/HP), chondroitin sulfate/dermatan sulfate (CS/DS), keratan sulfate (KS), and hyaluronic acid or hyaluronan (HA) [3,4]. 

HS is the most abundant one among them, accounting for 50–90% of the total GAGs [5]. HS is a member of glycosaminoglycan, which is composed of unbranched negatively charged disaccharide units and facilitates several important biological processes in health and disease [6,7,8]. Heparan sulfate proteoglycans (HSPGs) are linear macromolecular substances consisting of a core protein and one or more HS glycosaminoglycan chains, located at the cell surface and within the extracellular matrix (ECM). There are three key enzymes, including sulfatase1 (Sulf1), sulfatase2 (Sulf2) and heparanase that can cleave the HS polymers, releasing smaller fragments from HSPG complexes.

Three main basement membrane (BM) HSPGs have been well characterized: Perlecan, Agrin and collagen XVIII. Perlecan is a modular proteoglycan with homology to growth factors, Collagen XVIII is a hybrid collagen-proteoglycan with multiple regions and Agrin is a large glycoprotein that is released from motor neurons [9,10].

HA is an unbranched, nonsuflated glycosaminoglycan that consists of repeating disaccharide units of *N*-acetyl glucosamine and D-glucuronic acid [11]. Three types of eukaryotic hyaluronan synthase (HAS) have been identified, namely HAS1, HAS2 and HAS3. Among them, HAS1 and HAS2 can promote the synthesis of high molecular weight (Mr) HA. CD44 is a transmembrane glycoprotein that acts as a HA receptor and is one a well-accepted cancer stem cell (CSC) surface markers.

Syndecans and glypicans are major core proteins. Syndecans [9] are single transmembrane domain proteins capable of carrying three to five heparan sulfate and chondroitin sulfate chains. It interacts with a large variety of ligands, including fibroblast growth factors (FGF), vascular endothelial growth factor (VEGF), transforming growth factor-beta (TGF-β), fibronectin and antithrombin-1. There are four types of syndecans in human beings, namely syndecan-1 to syndecan-4; syndecan-1 has been measured in studies [10].

Glycoproteins are glycoconjugates formed by the covalent attachment of branched oligosaccharide chains to polypeptide chains. In addition, the extracellular matrix also contains abundant adhesive glycoproteins and proteoglycans. These components contribute to the barrier function to control cell migration and metastasis.

### 2.2. Glycocalyx On Cancer Cell Surface

#### 2.2.1. Altered Glycosylation

The glycocalyx of cancer cell surface is unique with abundant glycosylation, including sialylation, fucosylation, *O*-glycan truncation, and *N*- and *O*-linked glycan branching [12]. 

Sialylation owing to altered glycosyltransferases in cancer cells is critical for cell recognition, cell adhesion, and cell signaling [6]. In colon, stomach, and ovarian cancer, the lactosaminic chains are usually terminated by a sialic acid [7]. SLex is another sialylated product, which can bind to selectin and regulate the metastatic cascade by facilitating cancer cells and platelets to arrest in the endothelium [8]. SLea is another sialylated antigen and it is now widely used in clinical diagnosis of pancreatic, colorectal, gastric, and biliary cancer [13]. Cancer cell sialylation also results in polysialic acid expression, which is associated with high-grade tumors [14].

Fucosylation is catalyzed by a wide range of fucosyltransferases and can be divided into two subtypes: Terminal fucosylation and core fucosylation [15]. Core fucosylation means addition of α1,6-fucose to the innermost GlcNAc residue of *N*-glycans. It has been reported that increased core fucosylation is associated with lung cancer and breast cancer and can be detected in the serum in the process of hepatocarcinogenesis [16,17]. In clinic, core fucosylation of α-fetoprotein has been considered as an early biomarker for hepatocellular carcinoma diagnosis [18].

The overexpression of truncated *O*-glycans is another feature of cancer cell glycocalyx. These aberrant glycocalyx result from the incomplete synthesis of *O*-glycans that show abnormal expression of shortened glycans, including disaccharide T antigen, monosaccharide GalNAc (Tn) and their sialylated forms STn [19]. STn, in particular, could be detected in most cancer cells, e.g. stomach, breast, bladder, ovary and pancreas, and is hidden in healthy tissues [12]. Moreover, increased level of STn has been reported to be correlated with increased cancer cell proliferation, migration, invasion, and decreased cell adhesion. Therefore, it has been designated as the key prognostic marker and a target for the design of anticancer vaccines [20]. The key enzyme that catalyzes the reaction of abnormal *O*-glycosylation is GalNAc transferases (ppGalNAcTs), the enzyme initiating the reaction and controlling the density and sites of *O*-glycan addition [21]. This enzyme can be often observed in cancer. 

In addition, branching *N*-glycans resulting from the overexpression of complex β 1,6-branched *N*-linked glycans is also observed in cancer cells. This is due to the increased activity of *N*-acetylglucosamine (GlcNAc) transferases (GnT-V), encoded by the mannoside acetyl-glucosaminyltransferase 5 (MGAT5) [22]. It has been demonstrated that the upregulation of MGAT5 in a lung epithelial cell line led to loss of contact inhibition, increased cell motility and tumor formation in athymic mice [23]. Interestingly, these branched *N*-glycans can be further modified, elongated, and are always terminated with sialic acid or fucose, until it encounters the enzyme GnT-III. GnT-III is encoded by MGAT3 and catalyses the addition of bisecting GlcNAc N-glycans in a β1,4-linkage, resulting in elongation of *N*-glycans stop. Therefore, GnT-III has been reported to be involved in the suppression of cancer metastasis [24]. 

Except for glycosylation, gene expressions of syndecans in cancer cells are also different from normal cells.

#### 2.2.2. Altered Syndecan Expression in Cancer

Altered syndecan-1 expression has been observed in several cancer cells, including colon carcinoma, glioblastoma, breast cancer and ovarian cancer. Badiola et al. [25] reported that fibrillar collagen receptor discoidin domain receptor 2 deficiencies in hepatic stellate cells resulted in syndecan-1 expression upregulation and colon carcinoma metastasis. In breast cancer, syndecan-1 played dual roles. On one hand, as a receptor for collagen, syndecan-1 can be regulated by tumor-associated collagen signature-3, which leads to decreased collagen alignment and increased death in breast cancer patients [26]. On the other hand, syndecan-1 stimulated by peroxisome proliferator receptor activator gamma acts as a tumor suppressor, triggering the apoptosis of breast cancer cells [27]. In glioblastoma patients, overexpression of syndecan-1 is induced by a secreted glycoprotein, YKL-40 [28]. Finally, in ovarian cancer, enhanced expression of syndecan-1 promotes metastasis by activating mitogen-activated protein kinase, ERK, and phosphatidylinositol (PI)-3 kinase/AKT signaling [29]. 

During cancer progression, syndecan-2 expression is also altered. For example, the expression of syndecan-2 can be upregulated by fibroblast growth factor (FGF)-2, and inhibited by a-melanocyte-stimulating hormone, which are associated with melanoma cells migration [30]. Moreover, the translocation of syndecan-2 to lipid rafts induced by tubulin polymerization can also regulate cancer cell migration [31]. In osteosarcoma cells, the expression of syndecan-2 is repressed by the high activity of the canonical Wnt/RhoA pathway [32]. Decreased syndecan-4 expression has been shown to be associated with enhanced M5 melanoma cell migration and weakened attachment of these cells to fibronectin [33]. 

## 3. Associations between the GCX and Cancer

### 3.1. Cell Migration and Metastasis

Tumor cells can migrate from one place to another. As the tumor grows, some cancer cells can fall off from the original tumor and spread to other sites through the blood or lymph system to form new tumors. This process is also known as metastasis, which is the main cause of death from cancer.

#### 3.1.1. HA

Substantial evidence that HA plays an important role in cancer cell metastasis and invasion has been provided over the past decade [11,34,35]. High molecular weight HA is thought to provide a hydrated matrix that forces gaps in the extracellular matrix (ECM), enabling tumor cells to migrate and metastasize to other tissues within the tumor environment [36,37]. Rudrabhatla et al. [38] compared the patterns of HA expression between B16-F1 and B16-F10 melanoma cells in vitro and in situ. They proposed that components of the tumor microenvironment (e.g., lactate) can induce melanoma cells to express HA and therefore acquire an aggressive phenotype based on the experimental results. Zhang et al. [39] isolated subsets of the B16-F1 mouse melanoma cell line which expressed either high (HA-H) or low (HA-L) amounts of hyaluronan on their surfaces by using flow cytometry. The result showed that the HA-H cells formed larger and more numerous lung metastases than an equivalent number of HA-L cells after tail vein injection, which suggests that cell surface HA may play a critical role in the process of tumor metastasis. 

Fieber et al. [40] showed the expression of metalloproteases MMP-9 and MMP-13 in Lewis Lung Carcinoma (3LL) cells and primary embryonic fibroblasts were strongly induced when the cells were exposed to HA oligosaccharides. This result suggested that HA degradation in tumors might promote invasion. We thus speculate that different types of tumor cell produce different responses.

Moreover, Udabage et al. [36] studied endogenous levels of mRNA for the various HA synthase and degradation isoforms that were quantitated in 10 different human breast cancer cell lines by using real-time and comparative reverse transcriptase-polymerase chain reaction (RT-PCR).The results determined that highly invasive cell lines preferentially expressed the HAS2 and hyaluronidase-2 (Hyal-2) isoforms, while less invasive cells expressed HAS3 and hyaluronidase-3 (Hyal-3). In addition, they proved that there is a correlation between elevated levels of HA synthesis, CD44 expression and cancer cell migration, consequently highlighting that HA metabolism plays a pivotal role in the aggressive breast cancer phenotype. Interestingly, overexpression of CD44, the receptor of HA in mammary carcinoma or melanoma cells, inhibits tumor growth and metastasis [35,41]. 

Naor et al. [42] investigated CD44 and tumor metastasis using the mouse malignant LB lymphoma cell line, showing that CD44 promotes metastasis. Such promotion has also been proved for breast cancer [43]. However, in a previous study, Lopez et al. [44] demonstrated that CD44 can inhibit metastasis in breast cancer. The reason is possibly because different investigators used different techniques and approaches. 

#### 3.1.2. HSPG

HSPGs have also been shown to play important roles in cell migration and metastasis [45,46]. Gastric cancer cell lines MKN28 lack endogenous human sulfatase1 (HSulf-1). Li et al. [47] restored HSulf-1 expression in MKN28 and suppressed canonical Wnt signaling. They found that Sulf-1 expression inhibits cell proliferation and invasion. Later, Peterson et al. [48] reported that the overexpression of Sulf2 in MDA-MB-231 cells inhibited breast cancer cell invasion and metastasis in vitro as well as in vivo. These may be attributed to the enhancement of the synthesis of HS. However, the amount of HS can also be affected by heparanase, an enzyme that catalyzes the cleavage of HS into some smaller pieces. It has been demonstrated that heparanase may play an important role in promoting many cancer cells’ metastasis [49,50,51,52,53]. There are a certain amount of sites within HS chains where heparanase cleavage of HS to large degradation fragments takes place (5-10 kDa or 10-20 sugar units) [49]. This cleavage of HS may increase the solubility of a variety of signaling molecules, as a result increasing their access to receptors and facilitating signal transduction [54]. Using real-time quantitative PCR, Koliopanos et al. [55] suggested that the overexpression of heparanase in human pancreatic cancers facilitates cancer cell invasion, and consequently enhances the metastatic potential of the tumors. Meanwhile, Elassal et al. [56] suggested that heparanase enhances hepatocellular carcinoma cell growth and invasion. There are also a large number of experiments showing that heparanse is related to cells metastasis of the bladder [53], cervix [57], colon [56], endometrium [58] and multiple myeloma [59].

Agrin is well expressed in a HCC cell line, MHCC-LM3. Moreover, Chakraborty et al. [60] showed that in a wound-healing assay, Agrin depletion severely reduced the migration of MHCC-LM3 cells. It has also been revealed that Agrin has high expression in Oral squamous cell carcinoma (OSCC), and Agrin siRNA knockdown promoted a decrease in OSCC cell migration [61]. In other words, Agrin may promote cell migration.

#### 3.1.3. Syndecans 

Syndecan is involved in the regulation of cell migration. Afratis et al. [62] demonstrated that syndecans and glypicans (cell-surface proteoglycans associated with heparan sulfate) can accelerate cell signaling, focal adhesion kinase phosphorylation, tumor growth and migration. Lebakken et al. [63] transfected mouse syndecan-1 cDNA into human Raji cells and suggested that cell spreading is mediated by the syndecan-1 core protein. Mikami et al. [64] showed that loss of syndecan-1 in esophageal squamous cell carcinomas may play an important role in cell invasion and metastasis, being closely associated with its malignant potential. The same conclusion that loss of syndecan-1 expression is a characteristic feature of high metastatic potential has also been proven to be applicable to human hepatocellular carcinoma (HCC) [65].

### 3.2. Tumor Cell Adhesion

There is evidence that HA can promote cell adhesion [11,66]. However, recently, Ween et al. [67] indicated that small HA oligomers can block human ovarian cancer cell lines adhesion to peritoneal cells. The reason is that HA oligomers compete with large HA polymers for CD44 binding, and as a result they can block HA binding to CD44 on the peritoneal cells. A similar phenomenon was observed by Takabe et al [68], who showed that overexpression of HAS3 increased the production of HA and decreased MV3 melanoma cell adhesion.

It has been demonstrated that HS participates in cancer cell adhesion as well. Recently, Takemoto et al. [69] suggested that the clustering of heparan sulfate induced by adhesamine promoted cell adhesion. Interestingly, Goldshmidt et al. [70] indicated that expression of surface-associated heparanase in nonadherent lymphoma cells induces early stages of cell adhesion and this adhesion is independent of its enzymatic activity. Levy-Adam et al. [71] demonstrated that heparanase facilitates cell adhesion and spreading by clustering of cell surface heparan sulfate proteoglycans, which is consistent with the observation by Takemoto et al. There also exist examples that show that Agrin is an important factor activating and coordinating cellular adhesion of HCC cancer cells and OSCC cells [60,61].

It is well known that Syndecans contribute unique functional activities to the process of cell-matrix adhesion and cell-cell adhesion [63,72,73]. Syndecan-1 in lymphoblastoid B cells or Multiple myeloma (MM) cells was reported to promote cell adhesion [63,74]. Lamorte et al. [75] came to the conclusion that by mediating cell-to-matrix interactions, syndecan-1 promoted cell adhesion and invasion into the extracellular matrix. This is due to the fact that the reduced adherence of syndecan-1 knocks multiple myeloma endothelial cells (MMECs) to Matrigel. In another study, Park et al. [76] investigated mRNA expression of each syndecan family member in several colon cancer cell lines, and found that the expression of syndecan-2 was increased, facilitating the adhesion of carcinoma cells to the ECM. This phenomenon was also observed in breast carcinoma [77,78]. 

Recently, Zhang et al. [79] investigated the adhesion of MDA-MB-231 tumor cells to microvessels with or without the presence of 1 μM Sphingosine-1-phosphate (S1P). The results showed that S1P protected the endothelial glycocalyx layer by increasing its thickness and inhibited MDA-MB-231 tumor cell adhesion to the microvessel wall. This study provided evidence of the protective role of the whole glycocalyx layer in tumor cell adhesion.

### 3.3. Tumorigenesis

Tumor growth is a blood-dependent process and cancer cells begin to promote angiogenesis early in tumorigenesis. The formation of new irregular blood vessels from a preexisting vascular network is a feature of tumor angiogenesis. This abnormal angiogenesis plays an important role in tumor growth, survival and metastasis of most solid tumors [80,81]. There are many factors that can regulate angiogenesis, including VEGF, platelet-derived growth factor (PDGF), and basic fibroblast growth factor [82].

#### 3.3.1. HSPG

Fuster et al. [83] showed that deleting *N*-acetyl glucosamine *N-*deacetylase/sulfotransferase1 (Ndst1), a key enzyme in the process of heparan sulfate synthesis, results in decreased tumor angiogenesis. Therefore, they concluded that heparan sulfate is necessary for tumor angiogenesis.

Narita et al. [84] showed that Sulf1 inhibits angiogenesis and tumorigenesis in vivo by injecting a poorly differentiated breast cancer cell line, MDA468, as well as an ovarian cancer cell line into mice for tumor xenograft experiments. On the contrary, Morimoto et al. [85] implicated that Sulf2 can promote angiogenesis in breast cancer. These angiogenesis effects were also observed by Zhu et al. [86] in two human breast cancer cell lines, MCF-7 and MDA-MB-231, and other studies in human hepatocellular [82,87], pancreatic [88] and non-small cell lung carcinoma [89]. The underlying mechanism is unclear. However, Chen et al. [82] demonstrated in a Sulf2 knockout mouse model that the expression of Sulf2 in tumor cells can enhance the angiogenic potency of endothelial cells and periostin (POSTN) is the an effector protein in SULF2-induced angiogenesis. 

With the exception of Ndst1, Sulf1, and Sulf2, heparanase is another heparan sulfate associated enzyme that can promote angiogenesis [50,70,90]. Elassal et al. [56] suggested that heparanase enhances angiogenesis in hepatocellular carcinoma cell (HCC), and Gohji et al. [53] demonstrated that the expression of heparanase is positively correlated with angiogenesis of bladder cancer. Moreover, Barash et al. [91] showed that heparanase in myeloma enhances myeloma progression via CXCL10 downregulation; they concluded that heparanase has pro-tumorigenic effects.

Moreover, Zhou et al. [92] found that perlecan HS promoted angiogenesis in vivo for the removal of perlecan HS side chains, and led to impaired FGF-2-mediated angiogenesis. In an immortalized cell line derived from Kaposi’s sarcoma, suppression of perlecan expression promoted angiogenesis in vivo through increased angiogenic growth factor diffusion [93]. However, Mongiat et al. [94] discovered that the C terminus of perlecan potently inhibited angiogenesis, which indicate that different fragments have different effects.

In a recent study, Chakraborty et al. [60] discovered that Agrin is overexpressed in HCC, and Agrin promotes liver carcinogenesis, both in vitro and in vivo. 

#### 3.3.2. HA

It has been reported that native HA inhibits angiogenesis in vivo and partial degradation of HA molecules promotes angiogenesis [34,95]. Therefore, in clinic, an increased level of hyaluronidase, especially hyaluronidase-1 (HYAL1), would be a reliable marker for several types of malignant tumor. Kosaki et al. [96] transfected a mammalian HA synthase (HSA2) into human HT1080 cells to control the production of HA at the genetic level. They found that increased production of HA facilitates anchorage-independent growth and tumorigenicity of the cells. However, excess HA limited angiogenesis and diminished apparent cellular growth, resulting in tumorigenesis suppression [97].

Du et al. [98] injected a variable number of human cells into nude mice to test their xenotumor abilities. They proved that CD44 is a robust marker for colorectal CSC and plays an important role in tumorigenesis. In addition, Yu et al. [99] suggested that CD44 promotes angiogenesis in mammary tumor; the mechanism is CD44-associated MMP-9 can activate latent TGF-β by cleaving its TGF-β latency-associated protein, thereby inducing angiogenesis. 

#### 3.3.3. Syndecan

There is evidence that syndecan-1 can modulate angiogenesis in vivo. Caroline et al. [100] showed that the absence of syndecan-1 resisted Wnt-1-induced tumorigenesis of mice mammary gland. In a later study, Maeda et al. [101] found that the expression of syndecan-1 by stromal fibroblasts could stimulate angiogenesis in human breast carcinoma in vivo. In addition, Lamorte et al. [75] compared the ability of human umbilical vein endothelial cells (HUVECs), bone marrow endothelial cells (BMECs), multiple myeloma endothelial cells (MMECs), and MMEC with syndecan-1 silence to form in vitro capillary-like structures and proved that the expression of syndecan-1 promotes in vitro angiogenesis. These suggest that different species have a different reaction to syndecan-1.

### 3.4. Tumor Growth 

#### 3.4.1. HA

Studies have revealed that the overproduction of HA molecules promotes tumor growth in fibrosarcoma, prostate and mammary carcinoma [11,102]. On the other hand, HA oligomers with low-molecular weight (LMW) inhibit tumor growth [103]. This concludes that the effect of HA on tumor growth is size dependent. Kosaki et al. [96] found that the increased production of HA by cancer cells may play a pivotal role in enhancing tumor growth in vivo. However, Xu et al. [104] examined the biological activity of a 42-amino acid peptide (designated as BH-P), which includes three HA binding motifs from human brain HA binding protein. They demonstrated that BH-P inhibits the proliferation of tumor cells and tumor growth in vivo, and provided evidence of the size-dependent effect of HA on tumor growth. 

#### 3.4.2. HSPG

A number of studies have substantiated that HS plays an important role in the process of tumor growth [83,105,106]. However, the roles of Sulf1 and Sulf2 in different types of tumor growth under different microenvironments are ambiguous. 

Nawroth et al. 88 showed that both Sulf1 and Sulf2 are negative regulators of tumorigenesis in human pancreatic adenocarcinoma tumors, and Dai et al. [107] provided the first direct evidence that Sulf-1 and Sluf-2 can suppress myeloma tumor growth in vivo. In a later study, He et al. [108] showed that the absence of Sulf1 in ovarian cancer cells promotes tumor growth by decreasing the expression of pro-apoptotic proteins, such as Bim, suggesting that Sulf1 has anti-tumor effects [84,108]. This was also observed by Li et al. [108] for gastric cancer.

However, interestingly, Lai et al. [109] demonstrated that in hepatocellular carcinomas (HCC) cells, Sulf2 up-regulates the expression of cell surface Glypican-3, which in turn mediates Sulf2 oncogenic function. This suggests that Sulf2 may play a tumor-promoting role. Capurro et al. [110] showed that glypican 3 promotes the in vivo and in vitro growth of HCC by stimulating canonical Wnt signaling.

Perlecan is another component that plays an important role in tumor growth [9,92,111]. Similarly, there are examples that the same HSPGs can have either tumor-suppressing or tumor-promoting effects. Mathiak et al. [112] provided the first evidence that perlecan may inhibit the growth and invasiveness of fibrosarcoma cells by using HT-1080, a human fibrosarcoma cell line. While in a later study, Sharma et al. [113] showed that perlecan can promote the growth of colon carcinoma cells.

Collagen XVIII, as well as perlecan, is another HSPG from basement membranes that also has bipolar activity. Its C-terminal fragment endostatin inhibits angiogenesis and tumor growth by restricting endothelial proliferation and migration and inducing apoptosis of endothelial cells, while it has the opposite effect with HS chains [9]. 

In a recent study, Rivera et al. [114] suggested that silencing Agrin in oral cancer cells results in an impairment of in vitro proliferative and invasive growth programs, which means that Agrin promotes tumor growth.

Being consistent with the promotive effect of heparinase on angiogenesis, Cohen et al. [90] overexpressed the human heparanase gene (H-hpa) in MCF-7 human breast cancer cells and used MRI to monitor the progression of tumor growth. They found that heparanase promotes tumor growth.

#### 3.4.3. Syndecans 

Syndecan-1 has been reported to play important roles in tumor growth [63,74]. Evidence suggests that syndecan-1 induced tumor growth may be attributed to its promotion of cancer cell proliferation [46,74].

It has been revealed in vitro that purified syndecan-1 ectodomain inhibits the growth of S115 tumor cells [72], an epithelial-derived tumor-cell line. In addition, Szarvas et al. [115] used immunohistochemistry and enzyme-linked immunosorbent assay (ELISA) to analyze the tissue expression level and serum concentrations of syndecan-1 (SDC1) in bladder cancer (BC) patients. It turned out that the highest SDC1 serum levels exist in metastatic BCs, which supported the hypothesis that the circulating SDC1 ectodomain exerts tumor-promoting effects on distant metastatic sites.

Microenvironments may be a key factor impacting the growth-promoting effect of soluble syndecan-1. For example, several studies found that syndecan-1 may promote myeloma tumor growth in vivo, but inhibit growth of both carcinoma and myeloma cells in vitro [51,74,116,117]. However, in another study, Maeda et al. [101] demonstrated that syndecan-1 expressed by stromal fibroblasts promotes carcinoma cell growth, both in vivo and in vitro.

The relationships between different components of glycocalyx and progression of cancer have been summarized in Table 1.

## 4. Possible Mechanisms

### 4.1. As a Protective Barrier 

Endothelial glycocalyx acts as a protective barrier [118], repelling negatively charged molecules, white and red blood cells, platelets, and macromolecules like low-density lipoprotein (LDL) [119]. On the other hand, glycocalyx in the extracellular matrix forms a 3-dimensional barrier structure (designated as basement membranes) that limits cell migration and metastasis [3]. Therefore, modification of the glycocalyx (HSPG in particular) in the tumor microenvironment by Sulf1, Sulf2, or heparanase affects cancer cell proliferation, signaling, invasion, and metastasis [54].

### 4.2. Growth Factor Storage and Signaling

Syndecans, the core protein backbone of the glycocalyx, has been shown to play an important role in growth factor presentation and receptor activation [120], which can affect cell proliferation. Recently, Paszek et al. [121] developed a spatial-temporal simulation that integrated the micro-mechanics of the cell, glycocalyx, and extracellular matrix with a simple chemical model of integrin activation and ligand interaction. They predicted that the glycocalyx mediates integrin-ligand interaction and may be a key regulator of integrin clustering. Previous studies has demonstrated that integrin clustering is necessary for adhesion complex formation, which can influence growth factor mediated regulation of cell cycle progression [122]. For example, the interaction among integrin linked kinase, and its binding partners PINCH and ILKBP is critical for their localizing to adhesion structures and growth factor-mediated S phase entry [123]. Very recently, Woods et al. [124] coated tumor cells with synthetic mucin-mimetic glycopolymers to make them display glycocalyx of various thickness; they found that a bulky glycocalyx fosters metastasis by promoting G1 cell cycle progression. These effects were associated with enhanced integrin adhesion assembly, integrin-FAK mechanosensing and Akt signaling. According to these reports, glycocalyx mainly affects integrin clustering and mature adhesion complex formation, which in turn enhances growth factor signaling, a phenotype that is associated with cancer. This theory has been tested by Paszek et al. [125] in their later study. The results reveal that glycocalyx facilitates integrin clustering by funnelling active integrins into adhesions and altering integrin state by applying tension to matrix-bond integrins. This process is independent of actomyosin contractility. Moreover, high expression of glycocalyx promotes non-transformed mammary cells focal adhesion assembly and enhances integrin-dependent growth factor signaling to keep cell growth and survival. In glioblastoma, overexpression of syndecan-1 leads to increased vascular growth factor (VEGF) signaling and enhanced angiogenesis via focal adhesion kinase (FAK) phosphorylation at tyrosine 397, extracellular signal-related kinase (ERK) pathway [28]. 

### 4.3. Mechanotransduction

Cancer cells are exposed to interstitial flow-induced shear stress due to the high leaky properties of the capillaries surrounding a tumor, and the shear force is critical for cancer cell progress (Figure 1). Hompland et al. [126] demonstrated that the outward interstitial fluid flow velocity at the tumor surface, which can be measured by dynamic contrast-enhanced magnetic resonance imaging (DCE-MRI) with gadolinium diethylene-thiamine penta-acetic acid (Gd-DTPA), negatively correlated with survival in human cervical carcinoma. In an animal model, Bockhorn et al. [127] found that interstitial flow was present and it is essential to identify the metastatic potential of renal cancer cell lines. 

Glycocalyx plays an important role in mechanotransduction of shear stress regulated endothelial cell motility and proliferation [128], nitric oxide (NO) production [129], and water flux through endothelial cell monolayer [130]. Recently, Liu et al. [131] investigated hemodynamic environmental alteration induced vascular remodeling in vitro by using a home-made apparatus to expose rat abdominal aorta to a sterile, flow, high pressure condition for 4 days. The results showed that after the integrity of glycocalyx was diminished by hyaluronidase, flow and high-pressure loading induced vascular structural and functional remodeling was eliminated. This study provided evidence of the mechanotransduction role of the endothelial glycocalyx at the tissue level.

In addition, Tarbell et al. [132] proved that glycocalyx can sense interstitial flow by using a mathematical model, which was consistent with the experimental studies obtained by Shi et al [133]. They embedded smooth muscle cells in 3D collagen and revealed that heparan sulfate proteoglycans act as a mechanosensor in interstitial flow induced cell migration to activate the FAK-ERK pathway and upregulate matrix metalloproteinase (MMP) expression. By using the same cell/collagen suspension model to mimic the 3D interstitial flow microenvironment, Qazi H et al. [134] observed that cancer cell glycocalyx mediates mechanotransduction in interstitial flow induced cell motility and metastasis by regulating MMP-1, MMP-2, CD44, and α3 integrin expression. This is the first study attempting to explain the involvement of glycocalyx in cancer invasion from a mechanotransduction point. Later, Qazi et al. [135] extended their study by knockdown HS synthetic enzyme NDST1 of the highly metastatic renal carcinoma cells (SN12L1) and comparing the invasion ability of parental and knockdown cells. The results show that flow enhanced invasion was suppressed in HS depletion cells. Furthermore, they injected parental or knockdown cells into kidney capsules in mice and observed a 95% reduction in metastasis from the NDST1 knockdown cells injected site to distant organs, compared to controls cells. These findings support the key role of the cancer cell glycocalyx in interstitial flow-induced metastasis.

Integrin-FAK signaling directs proliferation of metastatic cancer cells [136]. In another study, Chakraborty et al. [60] showed that Agrin may serve as a mechanotransduction signal, as it can activate the integrin-FAK pathway. In a later study, Chakraborty et al. [137] suggested that Agrin is a mechanotransducing signal activating Yes-associated protein (YAP) through the integrin-focal adhesion-Lrp4/MuSK receptor pathway and that it promotes oncogenesis through YAP-dependent transcription. These findings have been discussed elsewhere as well, highlighting that Agrin serves as a mechanotransduction signal to activate YAP by suppressing the Hippo pathway and stimulating integrin-focal adhesion (FA), thus promoting liver cancer development [138].

## 5. Glycocalyx-targeting Therapeutic Approaches

Knowledge of the roles of glycocalyx in cancer is helpful in discovering promising biomarkers for early diagnosis, prediction, and treatment of clinical cancer. 

It has been verified by Terkelsen T et al. [139] that *N-*glycans secreted by breast cancer could be associated with their patterns in serum. They suggested that profiling of *N-*glycans may serve as novel biomarkers to improve the diagnosis and prognosis of breast cancer. 

Very recently, by integrating glycoproteomics with a novel reverse phase glycoprotein array, Chen et al. [140] verified 20 new potential biomarkers in extracellular vesicles from breast cancer patients. The association between ABO blood groups and risk of occurrence of ovarian and vulvar cancer has been widely studied [141]. Detailed serological cancer markers with clinical applications can be found in another review by Pinho et al [12]. Herein, we mainly focus on the strategies of cancer therapy targeting HS, HA, and syndecan, as described in this paper. 

### 5.1. HS Targeting Therapy

The main HS targeting strategies in clinic are based on its key role in angiogenesis, a complicated process that involves endothelial cell proliferation, migration, and differentiation into a new vessel within the tumor microenvironment. Research has shown that HSPG serves as a co-receptor of angiogenic factors, presenting them to their specific tyrosine kinase signaling receptors and triggering downstream cascades to initiate angiogenesis [142]. 

HS mimic heparin was previously explored for its antitumor properties [143]. However, its serious side-effects related to anticoagulation properties cause bleeding. Another strategy of cancer therapy was proposed by Jayson et al. [144], who demonstrated the effectiveness of synthetic HS fragments of a defined structure in blocking angiogenesis, thus killing the tumor by stopping nutrient and oxygen supply. This therapy is based on the theory that the activation of angiogenic cytokines, including fibroblast growth factor 2 (FGF2), interleukin 8 (IL-8) and stromal-cell-derived factor 1α (SDF-1α), are all HS dependent. Therefore, the exogenous HS fragments act as a substitute of the endogenous HS, competing with the binding sites of angiogenic cytokines and making them invalid. 

In according with the same theory, the PG500 series, PG545 in particular, have been developed to target the inhibition of both angiogenesis and heparanase activity; it is undergoing formal preclinical development [145]. 

However, unspecific modes of targeting still exists in both heparin and other HS mimetic therapies. Antibody-based therapy is one of the fastest growing areas in medical oncology. In clinic, antibodies targeting epidermal growth factor receptor 2 (HER-2), EGF receptor, VEGF, and CD20 have been approved for the treatment of breast, colorectal cancer and aggressive B-cell lymphomas [146]. Smaller antibodies like single-chain variable fragment (scFv) antibodies are also being applied more due to their pharmacokinetic properties [147]. Van Kuppevelt et al. established the development and characterization of αHS, a epitope-specific HS antibody, to probe the structural diversity of HS in different tissues [148]. However, Christianson et al. [149] demonstrated that binding of αHS to HS of ECs as well as glioblastoma cells may unexpectedly activate p38 MAPK-dependent signaling, eliciting a pro-angiogenic response. More recently, Gao et al. [150] reported that HS20, a human monoclonal antibody against glypican-3, could inhibit hepatocellular carcinoma proliferation both in vitro and in nude mice by disrupting the interaction of Wnt3a and glypican-3 and blocking Wnt3a/β-catenin signaling.

### 5.2. Glypican-3 Targeting Therapy

Glypican-3 (GPC3) is overexpressed in HCC, and is a useful tumor marker for cancer diagnosis [109,110]. As a result, novel therapeutic approaches for HCC could be generated by targeting glypican-3. Studies have shown that humanized GC33 (hGC33), a humanized anti-GPC3 monoclonal antibody significantly inhibits the growth of GPC3-positive human HCC xenografts in SCID mice; the mechanism induces antibody-dependent cellular cytotoxicity (ADCC) [151]. Komor et al. [152] identified a GPC3 peptide vaccine, which induces peptide-reactive cytotoxic T lymphocytes (CTLs), and showed that CTLs significantly inhibit the growth of human HCC xenografts in NOD/SCID mice. 

In addition, Nakatsura et al. [153] reported that GPC3 is a novel tumor marker for human melanoma diagnosis, especially in early stages of the disorder. Another research has proved that the antitumor effect of therapy with embryonic stem cell–derived dendritic cells (ES-DC) genetically modified to express murine GPC3 [154]. The mechanism is that in vivo transfer of glypican-3-transfectant ES-DC (ES-DC-GPC3) elicit specific CTLs, a protective effect against ovalbumin-expressing tumor cells. 

With the exception of HCC and melanoma, GPC3 was also expressed in other human malignancies, and has been reviewed in another article [155]. 

### 5.3. HA Targeting Therapy

HA has been reviewed in the previous section; HA and its receptors (i.e., CD44), HA synthases (i.e., HAS1 and HAS2), and hyaluronidase (HYAL1, 2, 3) are all associated with tumor growth and progression. Therefore, several targeted approaches have been developed to target the HA family. The most famous may be 4-Methylumbelliferone (4-MU), an orally bioavailable dietary supplement and a well-studied inhibitor of HA synthesis [156]. Cells treated with 4-MU show halting of HA synthesis. This may be a result of the following four effects: First, a major source of HA synthesis UDP-glucuronic acid (UGA) was deprived. This process is catalyzed by an enzyme known as UDP-glucuronosyltransferases, which transfers UGA to 4-MU instead. Second, 4-MU was reported to downregulate HAS2 and HAS3 expression by 60-80% in some cancer cells [157]. Third, it showed an inhibitory effect on HA receptors CD44 and RHAMM [158], suggesting a feedback loop between HA synthesis and HA receptor expression. Last, 4-MU treatment may cause HA signaling pathways disruption, including downregulation of the phosphorylation of ErbB2, Akt and their downstream effectors MMP-2/MMP-9 and IL-8 [159]. Based on these effects, 4-MU has been widely investigated in a number of cultured tumor cells. Promising effects have been observed; they include tumor cell proliferation, motility and invasion suppression, focal adhesion loss, and tumor growth inhibition [160], which suggests that 4-MU has a huge potential for clinical translation. 

Interestingly, HA oligosaccharides (oHA) with length smaller than 10 disaccharide units have shown promise in inhibiting tumor growth in both the subcutaneous B 16-F10 murine melanoma model [161] and the malignant peripheral nerve sheath tumor model [162]. This effect may be attributed to a direct blocking of HA signaling through CD44 and its related receptor tyrosine kinase [161]. Before oHA is translated into clinic, pre-clinic tests must pay attention to developing a more reliable method to synthesize its defined length on an industrial scale, since oHA beyond 10 disaccharide units shows angiogenic and tumor-promoting activity. 

In contrast to targeting HA synthesis, CD44 as the primary HA receptor is another target for cancer therapy. Several approaches, including DNA vaccine injection [163], CD44 siRNA delivery [164], and anti-CD44 monoclonal antibody administration [165] have been tested in clinic trials; the high toxicity reported as a main adverse reaction, however, needs to be overcome. 

Considering the fact that Haase, HYAL-1 in particular, could be a prognostic indicator for cancer progression, a variety of Haase inhibitors have been developed. In a study of 21 inhibitors, *O*-sulfated HA (Sha) was found to be the most effective in HYAL-1 inhibition, and the inhibitory effect was determined by the presence of sulfate per se, not the degree of sulfation [166]. Moreover, the PI3 kinase/Akt pathway may be the major signaling target that Sha interrupted [166]. Its potential in controlling tumor growth and progression is appealing for clinical cancer research. 

### 5.4. Syndecan-1 Targeting Therapy

The role of syndecan-1 in cancer cell migration and tumor growth has been described in the previous section. Several syndecan-1 targeting approaches have been discussed. One strategy is to down-regulate the expression of syndecan-1 by zoledronate [167], a third generation bisphosphonate, and nimesulide, which is usually used as a COX-2 specific anti-inflammatory drug [168]. Moreover, blocking the pro-tumorigenic activity of syndecan-1 by its specific antibody, i.e., Nbt062 [169] and B-B4 mAb [170], is another good option in treating several cancers. It has been demonstrated that co-localization of syndecan-1 and integrin as well as their interactions is crucial to triggering downstream signal cascades regulating angiogenesis and tumor metastasis. Therefore, drugs that can interrupt syndecan-integrin interaction and downstream signaling inhibitors will be ideal for cancer therapy. These drugs includes synstatin [171], the recombinant cell binding domain-heparin bind domain polypeptide of fibronectin (CBD-HepII) [172], and AZD7762 (Chk1 and MEK1/2 inhibitors) [173]. Interestingly, shedding of syndecan-1 was reported to be positively correlated with cancer development in mice [174]. Thus, reduction of shedding syndecan-1 may be a novel therapeutic approach to treat cancer [175]. 

The targeting therapies described above are summarized in Table 2.

## 6. Summary and Future Directions

**Figure 2:**
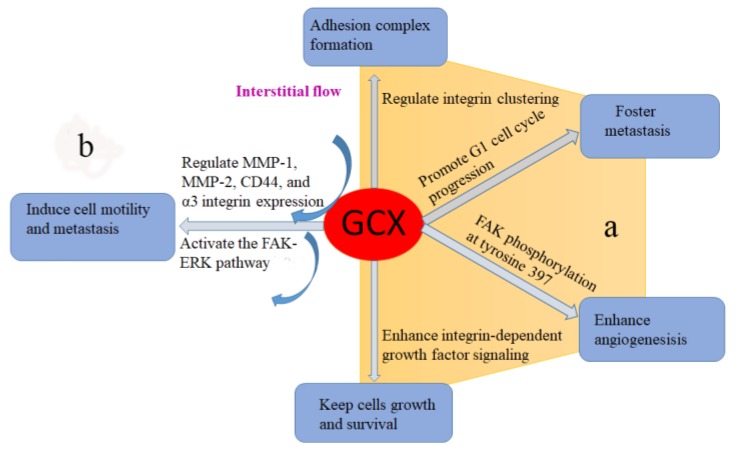
The involvement of cancer cell glycocalyx in tumor progression. (**a**) Glycocalyx enhances growth factor storage and signaling to regulate cancer cell adhesion, angiogenesis, metastasis, growth and survival. (**b**) Glycocalyx acts as a mechanotransducer of interstitial flow-induced shear stress to regulate cancer cell motility and metastasis.

Glycocalyx is a layer of multifunctional glycans located on the surfaces of a variety of cells, including vascular endothelial cells, smooth muscle cells, stem cells, epithelial, osteocytes, as well as cancer cells. It has been proved that glycocalyx on cancer cell surfaces showed different glycosylation and syndecan expressions, compared to vascular cells. However, it definitely plays important roles in cancer progression, including cell migration and metastasis, tumor cell adhesion, tumorigenesis, and tumor growth (Figure 2). The underlying mechanisms are unclear, but they could be associated with glycocalyx’s pivotal physiological role in growth factor storage and signaling; mechanotransduction; and as a protective barrier. Multiple approaches have been developed to target cancer cells’ glycocalyx. However, toxicity and specificity of these approaches require further optimization. In fact, cancer cells are exposed to interstitial flow-induced shear stress and this kind of shear force directly regulates the behavior of cancer cells (apoptosis vs. proliferation and migration). Investigating cancer cell glycocalyx, especially paying more attention to its mechanotransduction of interstitial flow induced shear stress, will be helpful in seeking promising therapeutic targets to kill tumors.

## Figures and Tables

**Table 1:** Relationship between components of glycocalyx and progression of cancer.

Component	Characteristics	Mechanisms in Cancer	References
HA	Unbranched, nonsuflated, repeating disaccharide units	Play dual roles in cell metastasis, adhesion, angiogenesis and tumor growth	[11,34,36,37,38,39,40,66,67,68,95,96,97,102,104]
Perlecan	Modular proteoglycan	Play dual roles in angiogenesis, tumor growth	[9,92,93,94,112,113]
Agrin	Released from motor neurons	Promote cell migration, tumorigenesis, tumor growth	[60,61,114]
Collagen XVIII	Hybrid collagen-proteoglycan	Play dual roles in angiogenesis, tumor growth	[9]
HS	Unbranched negatively charged disaccharide units	Promote cell metastasis, play dual roles in cell adhesion and tumor growth	[6,7,8,49,50,53,54,55,56,57,58,59,60,61,69,70,71,82,84,85,86,87,88,89,107,108,109,110]
Syndecans	Single transmembrane domain proteins	Play dual roles in metastasis, angiogenesis, tumor growth, promote cell adhesion	[9,25,26,27,28,29,51,62,63,64,65,72,73,74,75,76,77,78,100,101,115,116,117]

Abbreviations: HS: heparan sulfate; HA: hyaluronan.

**Table 2:** Glycocalyx-targeting therapeutic approaches.

	Targeting Therapy	Possible Mechanisms	Reference
HS	Synthetic HS fragments/PG500 series	Compete binding sites with endogenous HS and block angiogenesis	[144,145]
	Ahs	Antibodies specific for HS epitopes	[148]
	HS20	Disrupt the interaction of Wnt3a and glypican-3 and block Wnt3a/β-catenin signaling.	[150]
GPC3	hGC33	Induced ADCC	[151]
	GPC3 peptide vaccine	Induces peptide-reactive CTLs to inhibit growth of human HCC xenografts	[152]
	ES-DC	ES-DC-GPC3 elicit specific CTLs a protective effect against ovalbumin-expressing tumor cells	[154]
HA	4-MU	Compete UGA with HA	[156]
		Down-regulate HAS2 and HAS3 expression	[157]
		Inhibit HA receptor CD44 and RHAMM	[158]
		Disrupt HA signaling pathways	[159]
	oHA(smaller than 10 disaccharide units)	Block HA signaling	[161]
	sHA	Interrupt PI3 kinase/Akt pathway	[166]
Sdc1	Zoledronate	Disturb syndecan-1/integrins cross-talk	[167]
	Nimesulide	Down-regulate the expression of syndecan-1	[168]
	nBT062/ B-B4 mAb	Bind to similar or closely-related epitopes	[169,170]
	synstatin/ CBD-HepII	Interrupt syndecan-integrin interaction	[171,172]
	AZD7762	Interrupt downstream signaling inhibitors	[173]

Abbreviations: HS: heparan sulfate; HA: hyaluronan; 4-MU: 4-Methylumbelliferone; UGA: UDP-glucuronic acid; HAS2: hyaluronan synthase 2; HAS3: hyaluronan synthase 3; RHAMM: the Receptor for Hyaluronan-Mediated Motility; oHA: GPC3 glypican-3; hGC33 humanized GC33; ADCC antibody-dependent cellular cytotoxicity; CTLs cytotoxic T lymphocytes; HCC human hepatocellular carcinoma; ES-DC embryonic stem cell–derived dendritic cells; ES-DC-GPC3 glypican-3-transfectant ES-DC; HA oligosaccharides; sHA: *O-*sulfated HA; PI3: Phosphoinositide 3; Sdc1: syndecan-1; CBD-HepII: cell binding domain-heparin bind domain polypeptide of fibronectin.

## References

[1] Shurer C.R., Colville M.J., Gupta V.K., Head S.E., Kai F., Lakins J.N., Paszek M.J. (2018). Genetically encoded toolbox for glycocalyx engineering: Tunable control of cell adhesion, survival, and cancer cell behaviors. Acs. Biomater. Sci. Eng..

[2] Gasimli L., Linhardt R.J., Dordick J.S. (2012). Proteoglycans in stem cells. Biotechnol. Appl. Bioc..

[3] Tarbell J.M., Cancel L.M. (2016). The glycocalyx and its significance in human medicine. J. Intern. Med..

[4] Ly M., Laremore T.N., Linhardt R.J. (2010). Proteoglycomics: Recent progress and future challenges. Omics.

[5] Fu B.M.M., Tarbell J.M. (2013). Mechano-sensing and transduction by endothelial surface glycocalyx: Composition, structure, and function. Wires. Syst. Biol. Med..

[6] Kim Y.J., Varki A. (1997). Perspectives on the significance of altered glycosylation of glycoproteins in cancer. Glycoconj. J..

[7] Dall’Olio F., Chiricolo M. (2001). Sialyltransferases in cancer. Glycoconj. J..

[8] Nakamori S., Kameyama M., Imaoka S., Furukawa H., Ishikawa O., Sasaki Y., Kabuto T., Iwanaga T., Matsushita Y., Irimura T. (1993). Increased expression of sialyl lewisx antigen correlates with poor survival in patients with colorectal carcinoma: Clinicopathological and immunohistochemical study. Cancer Res..

[9] Iozzo R.V. (2005). Basement membrane proteoglycans: From cellar to ceiling. Nat. Rev. Mol. Cell Biol..

[10] Bezakova G., Ruegg M.A. (2003). New insights into the roles of agrin. Nat. Rev. Mol. Cell Biol..

[11] Adamia S., Maxwell C.A., Pilarski L.M. (2005). Hyaluronan and hyaluronan synthases: Potential therapeutic targets in cancer. Curr. Drug Targets Cardiovasc. Haematol. Disord..

[12] Pinho S.S., Reis C.A. (2015). Glycosylation in cancer: Mechanisms and clinical implications. Nat. Rev. Cancer.

[13] Locker G.Y., Hamilton S., Harris J., Jessup J.M., Kemeny N., Macdonald J.S., Somerfield M.R., Hayes D.F., Bast R.C. (2006). Asco 2006 update of recommendations for the use of tumor markers in gastrointestinal cancer. J. Clin. Oncol..

[14] Tanaka F., Otake Y., Nakagawa T., Kawano Y., Miyahara R., Li M., Yanagihara K., Inui K., Oyanagi H., Yamada T. (2001). Prognostic significance of polysialic acid expression in resected non-small cell lung cancer. Cancer Res..

[15] Carvalho A.S., Harduin-Lepers A., Magalhaes A., Machado E., Mendes N., Costa L.T., Matthiesen R., Almeida R., Costa J., Reis C.A. (2010). Differential expression of alpha-2,3-sialyltransferases and alpha-1,3/4-fucosyltransferases regulates the levels of sialyl lewis a and sialyl lewis x in gastrointestinal carcinoma cells. Int. J. Biochem. Cell Biol..

[16] Liu Y.C., Yen H.Y., Chen C.Y., Chen C.H., Cheng P.F., Juan Y.H., Khoo K.H., Yu C.J., Yang P.C., Hsu T.L. (2011). Sialylation and fucosylation of epidermal growth factor receptor suppress its dimerization and activation in lung cancer cells. Proc. Natl. Acad. Sci. USA.

[17] Potapenko I.O., Haakensen V.D., Luders T., Helland A., Bukholm I., Sorlie T., Kristensen V.N., Lingjaerde O.C., Borresen-Dale A.L. (2010). Glycan gene expression signatures in normal and malignant breast tissue; possible role in diagnosis and progression. Mol. Oncol..

[18] Hutchinson W.L., Du M.Q., Johnson P.J., Williams R. (1991). Fucosyltransferases: Differential plasma and tissue alterations in hepatocellular carcinoma and cirrhosis. Hepatology.

[19] Kudelka M.R., Ju T., Heimburg-Molinaro J., Cummings R.D. (2015). Simple sugars to complex disease-mucin-type o-glycans in cancer. Adv. Cancer Res..

[20] Julien S., Picco G., Sewell R., Vercoutter-Edouart A.S., Tarp M., Miles D., Clausen H., Taylor-Papadimitriou J., Burchell J.M. (2009). Sialyl-tn vaccine induces antibody-mediated tumour protection in a relevant murine model. Br. J. Cancer..

[21] Bennett E.P., Mandel U., Clausen H., Gerken T.A., Fritz T.A., Tabak L.A. (2012). Control of mucin-type o-glycosylation: A classification of the polypeptide galnac-transferase gene family. Glycobiology.

[22] Dennis J.W., Laferte S., Waghorne C., Breitman M.L., Kerbel R.S. (1987). Beta 1-6 branching of asn-linked oligosaccharides is directly associated with metastasis. Science.

[23] Demetriou M., Nabi I.R., Coppolino M., Dedhar S., Dennis J.W. (1995). Reduced contact-inhibition and substratum adhesion in epithelial cells expressing glcnac-transferase V. J. Cell Biol..

[24] Yoshimura M., Nishikawa A., Ihara Y., Taniguchi S., Taniguchi N. (1995). Suppression of lung metastasis of b16 mouse melanoma by n-acetylglucosaminyltransferase iii gene transfection. Proc. Natl. Acad. Sci. USA.

[25] Badiola I., Olaso E., Crende O., Friedman S.L., Vidal-Vanaclocha F. (2012). Discoidin domain receptor 2 deficiency predisposes hepatic tissue to colon carcinoma metastasis. Gut.

[26] Conklin M.W., Eickhoff J.C., Riching K.M., Pehlke C.A., Eliceiri K.W., Provenzano P.P., Friedl A., Keely P.J. (2011). Aligned collagen is a prognostic signature for survival in human breast carcinoma. Am. J. Pathol..

[27] Hu Y.P., Sun H.G., Owens R.T., Gu Z.N., Wu J.S., Chen Y.Q., O’Flaherty J.T., Edwards I.J. (2010). Syndecan-1-dependent suppression of pdk1/akt/bad signaling by docosahexaenoic acid induces apoptosis in prostate cancer. Neoplasia.

[28] Francescone R.A., Scully S., Faibish M., Taylor S.L., Oh D., Moral L., Yan W., Bentley B., Shao R. (2011). Role of ykl-40 in the angiogenesis, radioresistance, and progression of glioblastoma. J. Biochem. Physiol..

[29] Modrowski D., Orosco A., Thevenard J., Fromigue O., Marie P.J. (2005). Syndecan-2 overexpression induces osteosarcoma cell apoptosis: Implication of syndecan-2 cytoplasmic domain and jnk signaling. Bone.

[30] Lee J.H., Park H., Chung H., Choi S., Kim Y., Yoo H., Kim T.Y., Hann H.J., Seong I., Kim J. (2009). Syndecan-2 regulates the migratory potential of melanoma cells. J. Biochem. Physiol..

[31] Baljinnyam E., Iwatsubo K., Kurotani R., Wang X., Ulucan C., Iwatsubo M., Lagunoff D., Ishikawa Y. (2009). Epac increases melanoma cell migration by a heparan sulfate-related mechanism. Am. J. Physiol. Cell Physiol..

[32] Dieudonne F.X., Marion A., Hay E., Marie P.J., Modrowski D. (2010). High wnt signaling represses the proapoptotic proteoglycan syndecan-2 in osteosarcoma cells. Cancer Res..

[33] Chalkiadaki G., Nikitovic D., Berdiaki A., Sifaki M., Krasagakis K., Katonis P., Karamanos N.K., Tzanakakis G.N. (2009). Fibroblast growth factor-2 modulates melanoma adhesion and migration through a syndecan-4-dependent mechanism. Int. J. Biochem. Cell Biol..

[34] Toole B.P. (2004). Hyaluronan: From extracellular glue to pericellular cue. Nat. Rev. Cancer.

[35] Anttila M.A., Tammi R.H., Tammi M.I., Syrjanen K.J., Saarikoski S.V., Kosma V.M. (2000). High levels of stromal hyaluronan predict poor disease outcome in epithelial ovarian cancer. Cancer Res..

[36] Udabage L., Brownlee G.R., Nilsson S.K., Brown T.J. (2005). The over-expression of has2, hyal-2 and cd44 is implicated in the invasiveness of breast cancer. Exp. Cell Res..

[37] Weigel P.H., Hascall V.C., Tammi M. (1997). Hyaluronan synthases. J. Biochem. Physiol..

[38] Rudrabhatla S.R., Mahaffey C.L., Mummert M.E. (2006). Tumor microenvironment modulates hyaluronan expression: The lactate effect. J. Clin. Investig. Dermatol..

[39] Zhang L., Underhill C.B., Chen L. (1995). Hyaluronan on the surface of tumor cells is correlated with metastatic behavior. Cancer Res..

[40] Fieber C., Baumann P., Vallon R., Termeer C., Simon J.C., Hofmann M., Angel P., Herrlich P., Sleeman J.P. (2004). Hyaluronan-oligosaccharide-induced transcription of metalloproteases. J. Cell Sci..

[41] Toole B.P., Wight T.N., Tammi M.I. (2002). Hyaluronan-cell interactions in cancer and vascular disease. J. Biochem. Physiol..

[42] Naor D., Wallach-Dayan S.B., Zahalka M.A., Sionov R.V. (2008). Involvement of cd44, a molecule with a thousand faces, in cancer dissemination. Semin. Cancer Biol..

[43] Hill A., McFarlane S., Mulligan K., Gillespie H., Draffin J.E., Trimble A., Ouhtit A., Johnston P.G., Harkin D.P., McCormick D. (2006). Cortactin underpins cd44-promoted invasion and adhesion of breast cancer cells to bone marrowendothelial cells. Oncogene.

[44] Lopez J.I., Camenisch T.D., Stevens M.V., Sands B.J., McDonald J., Schroeder J.A. (2005). Cd44 attenuates metastatic invasion during breast cancer progression. Cancer Res..

[45] Bishop J.R., Schuksz M., Esko J.D. (2007). Heparan sulphate proteoglycans fine-tune mammalian physiology. Nature.

[46] Bernfield M., Gotte M., Park P.W., Reizes O., Fitzgerald M.L., Lincecum J., Zako M. (1999). Functions of cell surface heparan sulfate proteoglycans. Annu. Rev. Biochem..

[47] Li J., Mo M.L., Chen Z., Yang J., Li Q.S., Wang D.J., Zhang H., Ye Y.J., Li H.L., Zhang F. (2011). Hsulf-1 inhibits cell proliferation and invasion in human gastric cancer. Cancer Sci..

[48] Peterson S.M., Iskenderian A., Cook L., Romashko A., Tobin K., Jones M., Norton A., Gomez-Yafal A., Heartlein M.W., Concino M.F. (2010). Human sulfatase 2 inhibits in vivo tumor growth of mda-mb-231 human breast cancer xenografts. BMC Cancer.

[49] Vreys V., David G. (2007). Mammalian heparanase: What is the message?. J. Cell Mol. Med..

[50] Sanderson R.D., Yang Y., Suva L.J., Kelly T. (2004). Heparan sulfate proteoglycans and heparanase—Partners in osteolytic tumor growth and metastasis. Matrix Biol..

[51] Mali M., Andtfolk H., Miettinen H.M., Jalkanen M. (1994). Suppression of tumor cell growth by syndecan-1 ectodomain. J. Biochem. Physiol..

[52] Takaoka M., Naomoto Y., Ohkawa T., Uetsuka H., Shirakawa Y., Uno F., Fujiwara T., Gunduz M., Nagatsuka H., Nakajima M. (2003). Heparanase expression correlates with invasion and poor prognosis in gastric cancers. Lab. Investig..

[53] Gohji K., Hirano H., Okamoto M., Kitazawa S., Toyoshima M., Dong J., Katsuoka Y., Nakajima M. (2001). Expression of three extracellular matrix degradative enzymes in bladder cancer. Int. J. Cancer.

[54] Hammond E., Khurana A., Shridhar V., Dredge K. (2014). The role of heparanase and sulfatases in the modification of heparan sulfate proteoglycans within the tumor microenvironment and opportunities for novel cancer therapeutics. Front. Oncol..

[55] Koliopanos A., Friess H., Kleeff J., Shi X., Liao Q., Pecker I., Vlodavsky I., Zimmermann A., Buchler M.W. (2001). Heparanase expression in primary and metastatic pancreatic cancer. Cancer Res..

[56] El-Assal O.N., Yamanoi A., Ono T., Kohno H., Nagasue N. (2001). The clinicopathological significance of heparanase and basic fibroblast growth factor expressions in hepatocellular carcinoma. Clin. Cancer Res..

[57] Shinyo Y., Kodama J., Hongo A., Yoshinouchi M., Hiramatsu Y. (2003). Heparanase expression is an independent prognostic factor in patients with invasive cervical cancer. Ann. Oncol..

[58] Hasengaowa, Kodama J., Kusumoto T., Seki N., Matsuo T., Ojima Y., Nakamura K., Hongo A., Hiramatsu Y. (2006). Heparanase expression in both normal endometrium and endometrial cancer. Int. J. Gynecol. Cancer.

[59] Yang Y., MacLeod V., Bendre M., Huang Y., Theus A.M., Miao H.Q., Kussie P., Yaccoby S., Epstein J., Suva L.J. (2005). Heparanase promotes the spontaneous metastasis of myeloma cells to bone. Blood.

[60] Chakraborty S., Lakshmanan M., Swa H.L.F., Chen J.X., Zhang X.Q., Ong Y.S., Loo L.S., Akincilar S.C., Gunaratne J., Tergaonkar V. (2015). An oncogenic role of agrin in regulating focal adhesion integrity in hepatocellular carcinoma. Nat. Commun..

[61] Kawahara R., Granato D.C., Carnielli C.M., Cervigne N.K., Oliveria C.E., Rivera C., Yokoo S., Fonseca F.P., Lopes M., Santos-Silva A.R. (2014). Agrin and perlecan mediate tumorigenic processes in oral squamous cell carcinoma. PloS ONE.

[62] Afratis N., Gialeli C., Nikitovic D., Tsegenidis T., Karousou E., Theocharis A.D., Pavao M.S., Tzanakakis G.N., Karamanos N.K. (2012). Glycosaminoglycans: Key players in cancer cell biology and treatment. Febs. J..

[63] Lebakken C.S., Rapraeger A.C. (1996). Syndecan-1 mediates cell spreading in transfected human lymphoblastoid (raji) cells. J. Cell Biol..

[64] Mikami S., Ohashi K., Usui Y., Nemoto T., Katsube K., Yanagishita M., Nakajima M., Nakamura K., Koike M. (2001). Loss of syndecan-1 and increased expression of heparanase in invasive esophageal carcinomas. Jpn. J. Cancer Res..

[65] Matsumoto A., Ono M., Fujimoto Y., Gallo R.L., Bernfield M., Kohgo Y. (1997). Reduced expression of syndecan-1 in human hepatocellular carcinoma with high metastatic potential. Int. J. Cancer.

[66] Shea D.J., Li Y.W., Stebe K.J., Konstantopoulos K. (2017). E-selectin-mediated rolling facilitates pancreatic cancer cell adhesion to hyaluronic acid. FASEB J..

[67] Ween M.P., Hummitzsch K., Rodgers R.J., Oehler M.K., Ricciardelli C. (2011). Versican induces a pro-metastatic ovarian cancer cell behavior which can be inhibited by small hyaluronan oligosaccharides. Clin. Exp. Metastas..

[68] Takabe P., Bart G., Ropponen A., Rilla K., Tammi M., Tammi R., Pasonen-Seppanen S. (2015). Hyaluronan synthase 3 (has3) overexpression downregulates mv3 melanoma cell proliferation, migration and adhesion. Exp. Cell Res..

[69] Takemoto N., Suehara T., Frisco H.L., Sato S., Sezaki T., Kusamori K., Kawazoe Y., Park S.M., Yamazoe S., Mizuhata Y. (2013). Small-molecule-induced clustering of heparan sulfate promotes cell adhesion. J. Am. Chem Soc..

[70] Goldshmidt O., Zcharia E., Cohen M., Aingorn H., Cohen I., Nadav L., Katz B.Z., Geiger B., Vlodavsky I. (2003). Heparanase mediates cell adhesion independent of its enzymatic activity. FASEB J..

[71] Levy-Adam F., Feld S., Suss-Toby E., Vlodavsky I., Ilan N. (2008). Heparanase facilitates cell adhesion and spreading by clustering of cell surface heparan sulfate proteoglycans. PLoS ONE.

[72] Carey D.J. (1997). Syndecans: Multifunctional cell-surface co-receptors. Biochem. J..

[73] Beauvais D.M., Rapraeger A.C. (2004). Syndecans in tumor cell adhesion and signaling. Reprod. Biol. Endocrinol..

[74] Derksen P.W., Keehnen R.M., Evers L.M., van Oers M.H., Spaargaren M., Pals S.T. (2002). Cell surface proteoglycan syndecan-1 mediates hepatocyte growth factor binding and promotes met signaling in multiple myeloma. Blood.

[75] Lamorte S., Ferrero S., Aschero S., Monitillo L., Bussolati B., Omede P., Ladetto M., Camussi G. (2012). Syndecan-1 promotes the angiogenic phenotype of multiple myeloma endothelial cells. Leukemia.

[76] Park H., Kim Y., Lim Y., Han I., Oh E.S. (2002). Syndecan-2 mediates adhesion and proliferation of colon carcinoma cells. J. Biochem. Physiol..

[77] Beauvais D.M., Rapraeger A.C. (2003). Syndecan-1-mediated cell spreading requires signaling by alphavbeta3 integrins in human breast carcinoma cells. Exp. Cell Res..

[78] Lim H.C., Multhaupt H.A., Couchman J.R. (2015). Cell surface heparan sulfate proteoglycans control adhesion and invasion of breast carcinoma cells. Mol. Cancer.

[79] Zhang L., Zeng M., Fu B.M. (2017). Sphingosine-1-phosphate reduces adhesion of malignant mammary tumor cells mda-mb-231 to microvessel walls by protecting endothelial surface glycocalyx. Cell Mol. Biol..

[80] Carmeliet P., Jain R.K. (2000). Angiogenesis in cancer and other diseases. Nature.

[81] Kerbel R., Folkman J. (2002). Clinical translation of angiogenesis inhibitors. Nat. Rev. Cancer.

[82] Chen G., Nakamura I., Dhanasekaran R., Iguchi E., Tolosa E.J., Romecin P.A., Vera R.E., Almada L.L., Miamen A.G., Chaiteerakij R. (2017). Transcriptional induction of periostin by a sulfatase 2-tgfbeta1-smad signaling axis mediates tumor angiogenesis in hepatocellular carcinoma. Cancer Res..

[83] Fuster M.M., Wang L., Castagnola J., Sikora L., Reddi K., Lee P.H., Radek K.A., Schuksz M., Bishop J.R., Gallo R.L. (2007). Genetic alteration of endothelial heparan sulfate selectively inhibits tumor angiogenesis. J. Cell Biol..

[84] Narita K., Staub J., Chien J., Meyer K., Bauer M., Friedl A., Ramakrishnan S., Shridhar V. (2006). Hsulf-1 inhibits angiogenesis and tumorigenesis in vivo. Cancer Res..

[85] Morimoto-Tomita M., Uchimura K., Bistrup A., Lum D.H., Egeblad M., Boudreau N., Werb Z., Rosen S.D. (2005). Sulf-2, a proangiogenic heparan sulfate endosulfatase, is upregulated in breast cancer. Neoplasia.

[86] Zhu C.F., He L., Zhou X., Nie X., Gu Y. (2016). Sulfatase 2 promotes breast cancer progression through regulating some tumor-related factors. Oncol. Rep..

[87] Lai J.P., Sandhu D.S., Yu C.R., Han T., Moser C.D., Jackson K.K., Guerrero R.B., Aderca I., Isomoto H., Garrity-Park M.M. (2008). Sulfatase 2 up-regulates glypican 3, promotes fibroblast growth factor signaling, and decreases survival in hepatocellular carcinoma. Hepatology.

[88] Nawroth R., van Zante A., Cervantes S., McManus M., Hebrok M., Rosen S.D. (2007). Extracellular sulfatases, elements of the wnt signaling pathway, positively regulate growth and tumorigenicity of human pancreatic cancer cells. PLoS ONE.

[89] Lemjabbar-Alaoui H., van Zante A., Singer M.S., Xue Q., Wang Y.Q., Tsay D., He B., Jablons D.M., Rosen S.D. (2010). Sulf-2, a heparan sulfate endosulfatase, promotes human lung carcinogenesis. Oncogene.

[90] Cohen I., Pappo O., Elkin M., San T., Bar-Shavit R., Hazan R., Peretz T., Vlodavsky I., Abramovitch R. (2006). Heparanase promotes growth, angiogenesis and survival of primary breast tumors. Int. J. Cancer.

[91] Barash U., Zohar Y., Wildbaum G., Beider K., Nagler A., Karin N., Ilan N., Vlodavsky I. (2014). Heparanase enhances myeloma progression via cxcl10 downregulation. Leukemia.

[92] Zhou Z.J., Wang J.M., Cao R.H., Morita H., Soininen R., Chan K.M., Liu B., Cao Y.H., Tryggvason K. (2004). Impaired angiogenesis, delayed wound healing and retarded tumor growth in perlecan heparan sulfate-deficient mice. Cancer Res..

[93] Marchisone C., Del Grosso F., Masiello L., Prat M., Santi L., Noonan D.M. (2000). Phenotypic alterations in kaposi's sarcoma cells by antisense reduction of perlecan. Pathol. Oncol. Res..

[94] Mongiat M., Sweeney S.M., San Antonio J.D., Fu J., Iozzo R.V. (2003). Endorepellin, a novel inhibitor of angiogenesis derived from the c terminus of perlecan. J. Biol. Chem..

[95] West D.C., Kumar S. (1989). Hyaluronan and angiogenesis. Ciba. Found. Symp..

[96] Kosaki R., Watanabe K., Yamaguchi Y. (1999). Overproduction of hyaluronan by expression of the hyaluronan synthase has2 enhances anchorage-independent growth and tumorigenicity. Cancer Res..

[97] Bharadwaj A.G., Rector K., Simpson M.A. (2007). Inducible hyaluronan production reveals differential effects on prostate tumor cell growth and tumor angiogenesis. J. Biol. Chem..

[98] Du L., Wang H.Y., He L.Y., Zhang J.Y., Ni B.Y., Wang X.H., Jin H.J., Cahuzac N., Mehrpour M., Lu Y.Y. (2008). Cd44 is of functional importance for colorectal cancer stem cells. Clin. Cancer Res..

[99] Yu Q., Stamenkovic I. (2000). Cell surface-localized matrix metalloproteinase-9 proteolytically activates tgf-beta and promotes tumor invasion and angiogenesis. Genes Dev..

[100] Alexander C.M., Reichsman F., Hinkes M.T., Lincecum J., Becker K.A., Cumberledge S., Bernfield M. (2000). Syndecan-1 is required for wnt-1-induced mammary tumorigenesis in mice. Nat. Genet..

[101] Maeda T., Desouky J., Friedl A. (2006). Syndecan-1 expression by stromal fibroblasts promotes breast carcinoma growth in vivo and stimulates tumor angiogenesis. Oncogene.

[102] Llaneza A., Vizoso F., Rodriguez J.C., Raigoso P., Garcia-Muniz J.L., Allende M.T., Garcia-Moran M. (2000). Hyaluronic acid as prognostic marker in resectable colorectal cancer. Br. J. Surg..

[103] Spinelli F.M., Vitale D.L., Demarchi G., Cristina C., Alaniz L. (2015). The immunological effect of hyaluronan in tumor angiogenesis. Clin. Transl. Immunol..

[104] Xu X.M., Chen Y., Chen J., Yang S., Gao F., Underhill C.B., Creswell K., Zhang L. (2003). A peptide with three hyaluronan binding motifs inhibits tumor growth and induces apoptosis. Cancer Res..

[105] Belting M., Borsig L., Fuster M.M., Brown J.R., Persson L., Fransson L.A., Esko J.D. (2002). Tumor attenuation by combined heparan sulfate and polyamine depletion. Proc. Natl. Acad. Sci. USA.

[106] Esko J.D., Rostand K.S., Weinke J.L. (1988). Tumor formation dependent on proteoglycan biosynthesis. Science.

[107] Dai Y., Yang Y., MacLeod V., Yue X., Rapraeger A.C., Shriver Z., Venkataraman G., Sasisekharan R., Sanderson R.D. (2005). Hsulf-1 and hsulf-2 are potent inhibitors of myeloma tumor growth in vivo. J. Biochem. Physiol..

[108] He X., Khurana A., Roy D., Kaufmann S., Shridhar V. (2014). Loss of hsulf-1 expression enhances tumorigenicity by inhibiting bim expression in ovarian cancer. Int. J. Cancer.

[109] Lai J.P., Oseini A.M., Moser C.D., Yu C.R., Elsawa S.F., Hu C.L., Nakamura I., Han T., Aderca I., Isomoto H. (2010). The oncogenic effect of sulfatase 2 in human hepatocellular carcinoma is mediated in part by glypican 3-dependent wnt activation. Hepatology.

[110] Nakatsura T., Yoshitake Y., Senju S., Monji M., Komori H., Motomura Y., Hosaka S., Beppu T., Ishiko T., Kamohara H. (2003). Glypican-3, overexpressed specifically in human hepatocellular carcinoma, is a novel tumor marker. Biochem. Biophys. Res. Commun..

[111] Aviezer D., Hecht D., Safran M., Eisinger M., David G., Yayon A. (1994). Perlecan, basal lamina proteoglycan, promotes basic fibroblast growth factor-receptor binding, mitogenesis, and angiogenesis. Cell.

[112] Mathiak M., Yenisey C., Grant D.S., Sharma B., Iozzo R.V. (1997). A role for perlecan in the suppression of growth and invasion in fibrosarcoma cells. Cancer Res..

[113] Sharma B., Handler M., Eichstetter I., Whitelock J.M., Nugent M.A., Iozzo R.V. (1998). Antisense targeting of perlecan blocks tumor growth and angiogenesis in vivo. J. Clin. Investig..

[114] Rivera C., Zandonadi F.S., Sanchez-Romero C., Soares C.D., Granato D.C., Gonzalez-Arriagada W.A., Paes Leme A.F. (2018). Agrin has a pathological role in the progression of oral cancer. Br. J. Cancer.

[115] Szarvas T., Reis H., Kramer G., Shariat S.F., vom Dorp F., Tschirdewahn S., Schmid K.W., Kovalszky I., Rubben H. (2014). Enhanced stromal syndecan-1 expression is an independent risk factor for poor survival in bladder cancer. Hum. Pathol..

[116] Yang Y., Yaccoby S., Liu W., Langford J.K., Pumphrey C.Y., Theus A., Epstein J., Sanderson R.D. (2002). Soluble syndecan-1 promotes growth of myeloma tumors in vivo. Blood.

[117] Dhodapkar M.V., Abe E., Theus A., Lacy M., Langford J.K., Barlogie B., Sanderson R.D. (1998). Syndecan-1 is a multifunctional regulator of myeloma pathobiology: Control of tumor cell survival, growth, and bone cell differentiation. Blood.

[118] van den Berg B.M., Nieuwdorp M., Stroes E.S., Vink H. (2006). Glycocalyx and endothelial (dys) function: From mice to men. Pharmacol. Rep..

[119] Kang H., Fan Y., Sun A., Deng X. (2011). Compositional or charge density modification of the endothelial glycocalyx accelerates flow-dependent concentration polarization of low-density lipoproteins. Exp. Biol. Med..

[120] Lee D., Oh E.S., Woods A., Couchman J.R., Lee W. (1998). Solution structure of a syndecan-4 cytoplasmic domain and its interaction with phosphatidylinositol 4,5-bisphosphate. J. Biochem. Physiol..

[121] Paszek M.J., Boettiger D., Weaver V.M., Hammer D.A. (2009). Integrin clustering is driven by mechanical resistance from the glycocalyx and the substrate. PLos Comput. Biol..

[122] Walker J.L., Fournier A.K., Assoian R.K. (2005). Regulation of growth factor signaling and cell cycle progression by cell adhesion and adhesion-dependent changes in cellular tension. Cytokine Growth Factor Rev..

[123] Guo L., Wu C.Y. (2002). Regulation of fibronectin matrix deposition and cell proliferation by the pinch-ilk-ch-ilkbp complex. FASEB J..

[124] Woods E.C., Kai F., Barnes J.M., Pedram K., Pickup M.W., Hollander M.J., Weaver V.M., Bertozzi C.R. (2017). A bulky glycocalyx fosters metastasis formation by promoting g1 cell cycle progression. Elife.

[125] Paszek M.J., DuFort C.C., Rossier O., Bainer R., Mouw J.K., Godula K., Hudak J.E., Lakins J.N., Wijekoon A.C., Cassereau L. (2014). The cancer glycocalyx mechanically primes integrin-mediated growth and survival. Nature.

[126] Hompland T., Lund K.V., Ellingsen C., Kristensen G.B., Rofstad E.K. (2014). Peritumoral interstitial fluid flow velocity predicts survival in cervical carcinoma. Radiother Oncol..

[127] Bockhorn M., Roberge S., Sousa C., Jain R.K., Munn L.L. (2004). Differential gene expression in metastasizing cells shed from kidney tumors. Cancer Res..

[128] Yao Y., Rabodzey A., Dewey C.F. (2007). Glycocalyx modulates the motility and proliferative response of vascular endothelium to fluid shear stress. Am. J. Physiol. Heart Circ. Physiol..

[129] Ebong E.E., Lopez-Quintero S.V., Rizzo V., Spray D.C., Tarbell J.M. (2014). Shear-induced endothelial nos activation and remodeling via heparan sulfate, glypican-1, and syndecan-1. Integr. Biol..

[130] Williams D.A., Flood M.H. (2015). Capillary tone: Cyclooxygenase, shear stress, luminal glycocalyx, and hydraulic conductivity (lp). Physiol. Rep..

[131] Liu J., Kang H., Ma X., Sun A., Luan H., Deng X., Fan Y. (2018). Vascular cell glycocalyx-mediated vascular remodeling induced by hemodynamic environmental alteration. Hypertension.

[132] Tarbell J.M., Shi Z.D. (2013). Effect of the glycocalyx layer on transmission of interstitial flow shear stress to embedded cells. Biomech. Model. Mechan..

[133] Shi Z.D., Wang H., Tarbell J.M. (2011). Heparan sulfate proteoglycans mediate interstitial flow mechanotransduction regulating mmp-13 expression and cell motility via fak-erk in 3d collagen. PLoS ONE.

[134] Qazi H., Palomino R., Shi Z.D., Munn L.L., Tarbell J.M. (2013). Cancer cell glycocalyx mediates mechanotransduction and flow-regulated invasion. Integr. Biol..

[135] Qazi H., Shi Z.D., Song J.W., Cancel L.M., Huang P., Zeng Y., Roberge S., Munn L.L., Tarbell J.M. (2016). Heparan sulfate proteoglycans mediate renal carcinoma metastasis. Int. J. Cancer.

[136] Shibue T., Weinberg R.A. (2009). Integrin beta1-focal adhesion kinase signaling directs the proliferation of metastatic cancer cells disseminated in the lungs. Proc. Natl. Acad. Sci. USA.

[137] Chakraborty S., Njah K., Pobbati A.V., Lim Y.B., Raju A., Lakshmanan M., Tergaonkar V., Lim C.T., Hong W.J. (2017). Agrin as a mechanotransduction signal regulating yap through the hippo pathway. Cell Rep..

[138] Xiong W.C., Mei L. (2017). Agrin to yap in cancer and neuromuscular junctions. Trends Cancer.

[139] Terkelsen T., Haakensen V.D., Saldova R., Gromov P., Hansen M.K., Stockmann H., Lingjaerde O.C., Borresen-Dale A.L., Papaleo E., Helland A. (2018). *N-*glycan signatures identified in tumor interstitial fluid and serum of breast cancer patients: Association with tumor biology and clinical outcome. Mol. Oncol..

[140] Chen I.H., Aguilar H.A., Paez J.S.P., Wu X.F., Pan L., Wendt M.K., Iliuk A.B., Zhang Y., Tao W.A. (2018). Analytical pipeline for discovery and verification of glycoproteins from plasma-derived extracellular vesicles as breast cancer biomarkers. Anal. Chem..

[141] Sartorius C.M., Schoetzau A., Kettelhack H., Fink D., Hacker N.F., Fedier A., Jacob F., Heinzelmann-Schwarz V. (2018). Abo blood groups as a prognostic factor for recurrence in ovarian and vulvar cancer. PLoS ONE.

[142] Belting M. (2003). Heparan sulfate proteoglycan as a plasma membrane carrier. Trends Biochem. Sci..

[143] Parish C.R., Freeman C., Brown K.J., Francis D.J., Cowden W.B. (1999). Identification of sulfated oligosaccharide-based inhibitors of tumor growth and metastasis using novel in vitro assays for angiogenesis and heparanase activity. Cancer Res..

[144] Jayson G.C., Miller G.J., Hansen S.U., Barath M., Gardiner J.M., Avizienyte E. (2014). The development of anti-angiogenic heparan sulfate oligosaccharides. Biochem Soc. Trans..

[145] Dredge K., Hammond E., Davis K., Li C.P., Liu L., Johnstone K., Handley P., Wimmer N., Gonda T.J., Gautam A. (2010). The pg500 series: Novel heparan sulfate mimetics as potent angiogenesis and heparanase inhibitors for cancer therapy. Investig. New Drug.

[146] Scott A.M., Wolchok J.D., Old L.J. (2012). Antibody therapy of cancer. Nat. Rev. Cancer.

[147] Accardi L., Di Bonito P. (2010). Antibodies in single-chain format against tumour-associated antigens: Present and future applications. Curr. Med. Chem..

[148] Van Kuppevelt T.H., Dennissen M.A., van Venrooij W.J., Hoet R.M., Veerkamp J.H. (1998). Generation and application of type-specific anti-heparan sulfate antibodies using phage display technology. Further evidence for heparan sulfate heterogeneity in the kidney. J. Biochem. Physiol..

[149] Christianson H.C., van Kuppevelt T.H., Belting M. (2012). Scfv anti-heparan sulfate antibodies unexpectedly activate endothelial and cancer cells through p38 mapk: Implications for antibody-based targeting of heparan sulfate proteoglycans in cancer. PLoS ONE.

[150] Gao W., Kim H., Feng M.Q., Phung Y., Xavier C.P., Rubin J.S., Ho M. (2014). Inactivation of wnt signaling by a human antibody that recognizes the heparan sulfate chains of glypican-3 for liver cancer therapy. Hepatology.

[151] Ishiguro T., Sugimoto M., Kinoshita Y., Miyazaki Y., Nakano K., Tsunoda H., Sugo I., Ohizumi I., Aburatani H., Hamakubo T. (2008). Anti-glypican 3 antibody as a potential antitumor agent for human liver cancer. Cancer Res..

[152] Komori H., Nakatsura T., Senju S., Yoshitake Y., Motomura Y., Ikuta Y., Fukuma D., Yokomine K., Harao M., Beppu T. (2006). Identification of hla-a2-or hla-a24-restricted ctl epitopes possibly useful for glypican-3-specific immunotherapy of hepatocellular carcinoma. Clin. Cancer Res..

[153] Nakatsura T., Kageshita T., Ito S., Wakamatsu K., Monji M., Ikuta Y., Senju S., Ono T., Nishimura Y. (2004). Identification of glypican-3 as a novel tumor marker for melanoma. Clin. Cancer Res..

[154] Motomura Y., Senju S., Nakatsura T., Matsuyoshi H., Hirata S., Monji N., Komori H., Fukuma D., Baba H., Nishimura Y. (2006). Embryonic stem cell-derived dendritic cells expressing glypican-3, a recently identified oncofetal antigen, induce protective immunity against highly metastatic mouse melanoma, b16-f10. Cancer Res..

[155] Ho M., Kim H. (2011). Glypican-3: A new target for cancer immunotherapy. Eur. J. Cancer.

[156] Lokeshwar V.B., Mirza S., Jordan A. (2014). Targeting hyaluronic acid family for cancer chemoprevention and therapy. Adv. Cancer Res..

[157] Kultti A., Pasonen-Seppanen S., Jauhiainen M., Rilla K.J., Karna R., Pyoria E., Tammi R.H., Tammi M.I. (2009). 4-methylumbelliferone inhibits hyaluronan synthesis by depletion of cellular udp-glucuronic acid and downregulation of hyaluronan synthase 2 and 3. Exp. Cell Res..

[158] Lokeshwar V.B., Lopez L.E., Munoz D., Chi A., Shirodkar S.P., Lokeshwar S.D., Escudero D.O., Dhir N., Altman N. (2010). Antitumor activity of hyaluronic acid synthesis inhibitor 4-methylumbelliferone in prostate cancer cells. Cancer Res..

[159] Urakawa H., Nishida Y., Wasa J., Arai E., Zhuo L., Kimata K., Kozawa E., Futamura N., Ishiguro N. (2012). Inhibition of hyaluronan synthesis in breast cancer cells by 4-methylumbelliferone suppresses tumorigenicity in vitro and metastatic lesions of bone in vivo. Int. J. Cancer.

[160] Uchakina O.N., Ban H., McKallip R.J. (2013). Targeting hyaluronic acid production for the treatment of leukemia: Treatment with 4-methylumbelliferone leads to induction of mapk-mediated apoptosis in k562 leukemia. Leuk. Res..

[161] Ghatak S., Misra S., Toole B.P. (2002). Hyaluronan oligosaccharides inhibit anchorage-independent growth of tumor cells by suppressing the phosphoinositide 3-kinase/akt cell survival pathway. J. Biochem. Physiol..

[162] Slomiany M.G., Dai L., Bomar P.A., Knackstedt T.J., Kranc D.A., Tolliver L., Maria B.L., Toole B.P. (2009). Abrogating drug resistance in malignant peripheral nerve sheath tumors by disrupting hyaluronan-cd44 interactions with small hyaluronan oligosaccharides. Cancer Res..

[163] Pilon-Thomas S., Verhaegen M., Kuhn L., Riker A., Mule J.J. (2006). Induction of anti-tumor immunity by vaccination with dendritic cells pulsed with anti-cd44 igg opsonized tumor cells. Cancer Immunol. Immunother..

[164] Shah V., Taratula O., Garbuzenko O.B., Taratula O.R., Rodriguez-Rodriguez L., Minko T. (2013). Targeted nanomedicine for suppression of cd44 and simultaneous cell death induction in ovarian cancer: An optimal delivery of sirna and anticancer drug. Clin. Cancer Res..

[165] Rajasagi M., von Au A., Singh R., Hartmann N., Zoller M., Marhaba R. (2010). Anti-cd44 induces apoptosis in t lymphoma via mitochondrial depolarization. J. Cell Mol. Med..

[166] Isoyama T., Thwaites D., Selzer M.G., Carey R.I., Barbucci R., Lokeshwar V.B. (2006). Differential selectivity of hyaluronidase inhibitors toward acidic and basic hyaluronidases. Glycobiology.

[167] Dedes P.G., Gialeli C., Tsonis A.I., Kanakis I., Theocharis A.D., Kletsas D., Tzanakakis G.N., Karamanos N.K. (2012). Expression of matrix macromolecules and functional properties of breast cancer cells are modulated by the bisphosphonate zoledronic acid. Biochim. Biophys. Acta.

[168] Paul A.G., Sharma-Walia N., Chandran B. (2011). Targeting kshv/hhv-8 latency with cox-2 selective inhibitor nimesulide: A potential chemotherapeutic modality for primary effusion lymphoma. PLoS ONE.

[169] Ikeda H., Hideshima T., Fulciniti M., Lutz R.J., Yasui H., Okawa Y., Kiziltepe T., Vallet S., Pozzi S., Santo L. (2009). The monoclonal antibody nbt062 conjugated to cytotoxic maytansinoids has selective cytotoxicity against cd138-positive multiple myeloma cells in vitro and in vivo. Clin. Cancer Res..

[170] Rousseau C., Ruellan A.L., Bernardeau K., Kraeber-Bodere F., Gouard S., Loussouarn D., Sai-Maurel C., Faivre-Chauvet A., Wijdenes J., Barbet J. (2011). Syndecan-1 antigen, a promising new target for triple-negative breast cancer immuno-pet and radioimmunotherapy. A preclinical study on mda-mb-468 xenograft tumors. EJNMMI Res..

[171] Beauvais D.M., Ell B.J., McWhorter A.R., Rapraeger A.C. (2009). Syndecan-1 regulates alphavbeta3 and alphavbeta5 integrin activation during angiogenesis and is blocked by synstatin, a novel peptide inhibitor. J. Exp. Med..

[172] Gong W., Liu Y., Huang B., Lei Z., Wu F.H., Li D., Feng Z.H., Zhang G.M. (2008). Recombinant cbd-hepii polypeptide of fibronectin inhibits alphavbeta3 signaling and hematogenous metastasis of tumor. Biochem. Biophys. Res. Commun..

[173] Pei X.Y., Dai Y., Youssefian L.E., Chen S., Bodie W.W., Takabatake Y., Felthousen J., Almenara J.A., Kramer L.B., Dent P. (2011). Cytokinetically quiescent (g0/g1) human multiple myeloma cells are susceptible to simultaneous inhibition of chk1 and mek1/2. Blood.

[174] Ramya D., Siddikuzzaman, Grace V.M. (2012). Effect of all-trans retinoic acid (atra) on syndecan-1 expression and its chemoprotective effect in benzo(alpha)pyrene-induced lung cancer mice model. Immunopharmacol. Immunotoxicol..

[175] Choi S., Kang D.H., Oh E.S. (2013). Targeting syndecans: A promising strategy for the treatment of cancer. Expert Opin. Ther. Targets.

